# 14 Juvenile idiopathic arthritis: osteoporosis and fractures

**DOI:** 10.1093/rheumatology/keac496.010

**Published:** 2022-10-07

**Authors:** Ahlem Ben Ammou, Safa Rahmouni, Soumaya Boussaid, Houssem Tbini, Samia Jemmali, Sonia Rekik, Khaoula Zouaoui, Hela Sahli, Mohammed Elleuch

**Affiliations:** 1 Department of Rheumatology, La Rabta Hospital, Tunis, Tunisia; 2 University of Tunis El Manar

## Abstract

**Background:**

Juvenile idiopathic arthritis (JIA) is the most common inflammatory arthritis in children. Chronic inflammation, as well as the various treatments used during JIA increase the risk of occurrence of osteoporosis (OP) and fractures.

**Objectives:**

The objective of this work was to determine the frequency of OP in JIA and to investigate the factors associated with its occurrence.

**Methods:**

This was a retrospective study including adults with long-standing JIA according to the International League of Associations for Rheumatology (ILAR) criteria over a period of 28 years (1994–2022). We collected sociodemographic and anthropometric parameters, clinical data, results of biological assessments, bone densitometry results and data on prescribed therapies. We compared these variables according to the bone densitometry profile to assess the factors associated with OP in JIA.

**Results:**

There were 29 patients (17 females and 12 males), the mean age was 35.69 ± 11.72 [18–61] years. The mean age of disease onset was 11.10 ± 4.25 [2–16] years. The average diagnostic delay was 52.96 ± 95.97 [0–336] months. The average disease duration was 24.48 ± 12.76 [1–47] years. Three patients were smokers. The mean BMI was 21.20 ± 4.88 [14.17–27.55] kg/m2, and 4 patients had a BMI ≥ 25 kg/m².

The polyarticular form was the most frequent, noted in 55.2% of cases (*n* = 16). NSAIDs, corticosteroids and methotrexate were prescribed in 62.1%, 69% and 79.3% of cases respectively.

OP was found in 24.1% of cases (*n* = 7). Mean T-score values at the lumbar and femoral sites were −2.20 ± 1.22 SD and −2.31 ± 0.97 SD, respectively. Six patients suffered at least one fracture.

Patients with OP had lower BMI than patients with normal bone density (17.02 ± 2.46 *vs* 23.91 ± 5.42; *p* = 0.046). Furthermore, absence of rheumatoid factor was associated with OP in our study (83.3% *vs* 16.7%; *p* = 0.008).

On the other hand, the following parameters were not associated with the occurrence of OP in our study: age, gender, smoking, age at onset and duration of progression of JIA, BMI, extra-articular manifestations, CRP, antinuclear antibodies, ACPA and erosive character. Regarding the treatment received (NSAIDs, corticosteroids and methotrexate), no difference was found between patients.

**Conclusion:**

Osteoporosis in JIA is common and has a prognostic impact. It must be systematically screened throughout the follow-up. In our study, OP was associated with the absence of rheumatoid factor and was more frequent in patients with low BMI.

